# Supplementary material for: Tau activates microglia via the PQBP1-cGAS-STING pathway to promote brain inflammation
Source: Nat Commun. 2021 Nov 15;12:6565. doi: 10.1038/s41467-021-26851-2 (PMC8592984; doi:10.1038/s41467-021-26851-2)
Supplement: Supplementary file 1 — Supplementary Information [file 41467_2021_26851_MOESM1_ESM.pdf]

Tau activates microglia via the PQBP1-cGAS-STING pathway to promote brain inflammation

Meihua Jin[1,$], Hiroki Shiwaku[2,$], Hikari Tanaka[1,$], Takayuki Obita[3], Sakurako Ohuchi[3], Yuki Yoshioka[1], Xiaocen Jin[1], Kanoh Kondo[1], Kyota Fujita[1], Hidenori Homma[1], Kazuyuki Nakajima[4], Mineyuki Mizuguchi[3] and Hitoshi Okazawa[1,#]

[1]Department of Neuropathology, Medical Research Institute and Center for Brain Integration Research, Tokyo Medical and Dental University, 1-5-45 Yushima, Bunkyo-ku, Tokyo 113-8510, Japan.

[2]Department of Psychiatry, Graduate School of Medical and Dental Sciences, Tokyo Medical and Dental University, 1-5-45 Yushima, Bunkyo-ku, Tokyo 113-8510, Japan.

[3]Faculty of Pharmaceutical Sciences, Graduate School of Innovative Life Science, University of Toyama; 2630, Sugitani, Toyama 930-0194, Japan.

[4]Department of Bioinformatics, Institute of Bioinformatics, Soka university, 1-236 Tangi-machi, Hachioji, Tokyo 192-8577, Japan

$ These authors contributed equally

# Corresponding author

okazawa-tky@umin.ac.jp

Supplementary Figure 1

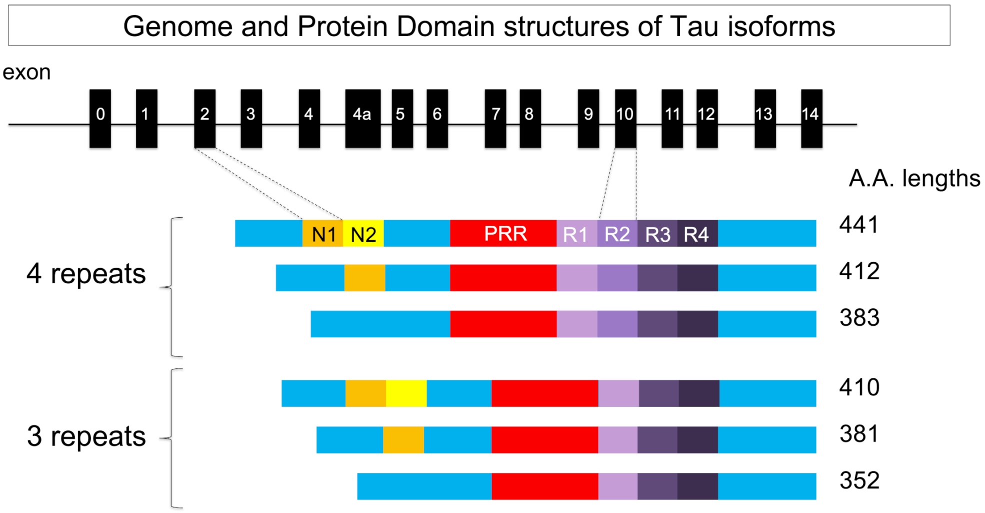

Supplementary Figure 1
Genome and protein domain structures of Tau isoforms
N1/2, N-terminal domain; R1/2/3/4, C-terminal microtubule-binding domain (tau repeat domain); PRR, proline-rich region.

Supplementary Figure 2

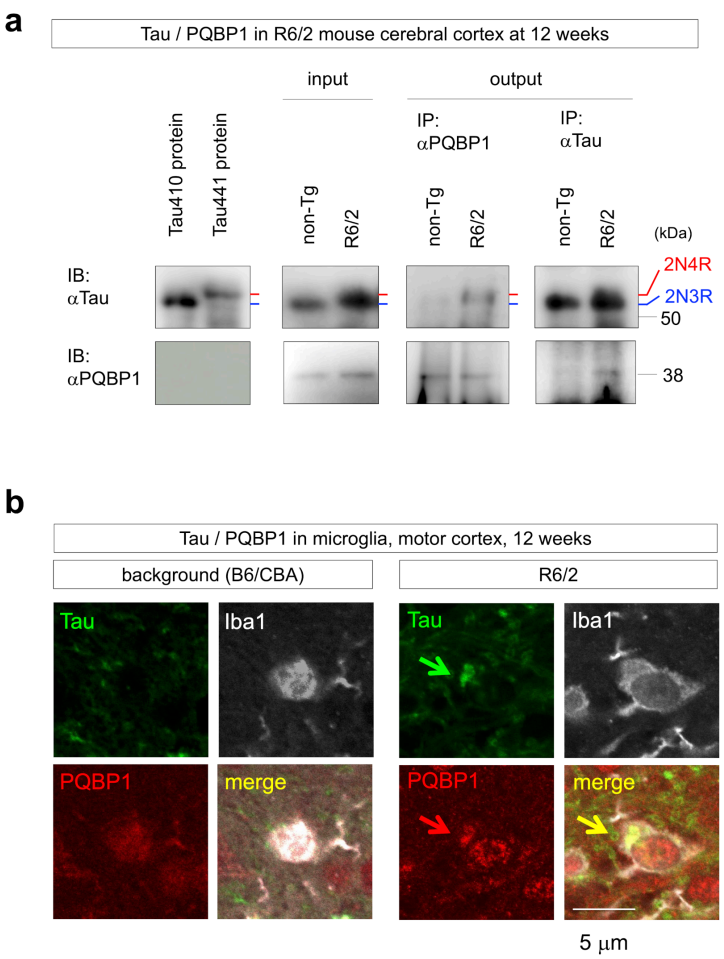

Supplementary Figure 2
In vivo interaction between PQBP1 and Tau in R6/2 mice
a) Immunoprecipitation revealed interaction between PQBP1 and Tau in cerebral cortex tissues prepared from R6/2 Huntington's disease model mice. IB, immunoblot.
b) Immunohistochemistry revealed colocalization of PQBP1 and tau in brain microglia of R6/2 mice at the age of 12 weeks.
Results in (a) and (b) are representative of three independent repeat experiments.

Supplementary Figure 3

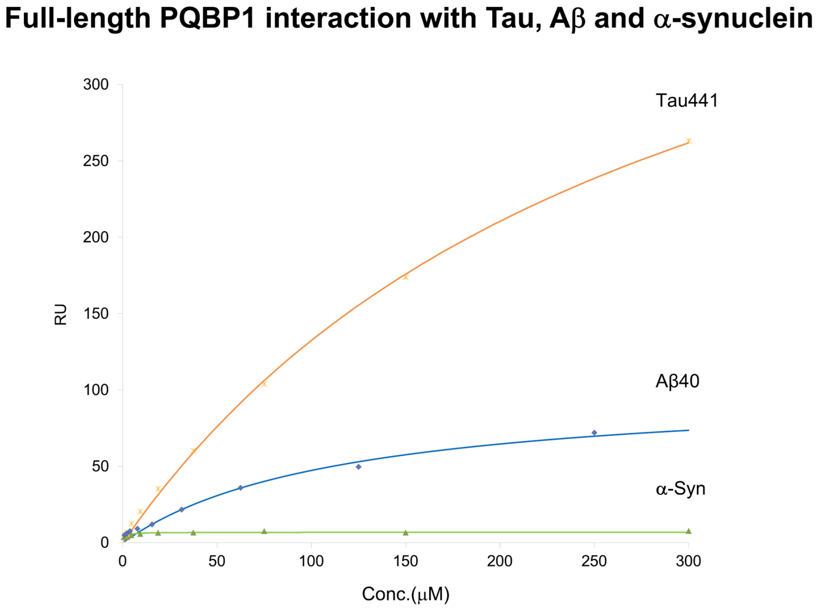

Supplementary Figure 3
Comparison of binding properties of Tau, Aβ and α-Synuclein to PQBP1
SPR analysis of interactions of full-length PQBP1 to the immobilized Tau 441 (red), Aβ (blue), and α-Synuclein (green). The binding response at equilibrium was plotted against the concentration of PQBP1. RU, resonance unit.

Supplementary Figure 4

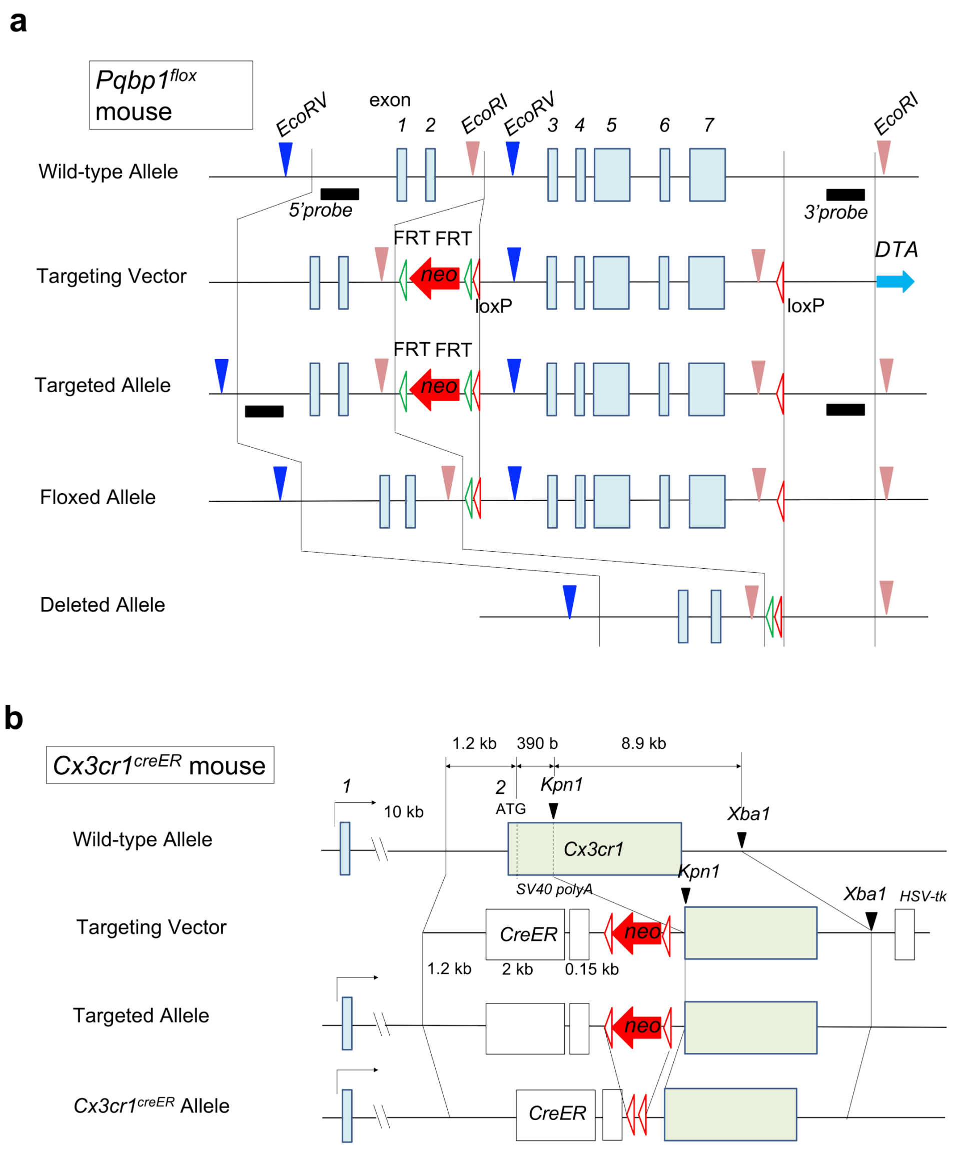

Supplementary Figure 4
Generation of tamoxifen-inducible and microglia-specific *Pqbp1*-cKO mouse
a) Generation method of *Pqbp1^{flox}* mouse
FRT, flippase recognition target; DTA, diphtheria toxin A.
b) Generation method of *Cx3cr1^{creER}* mouse

Supplementary Figure 5

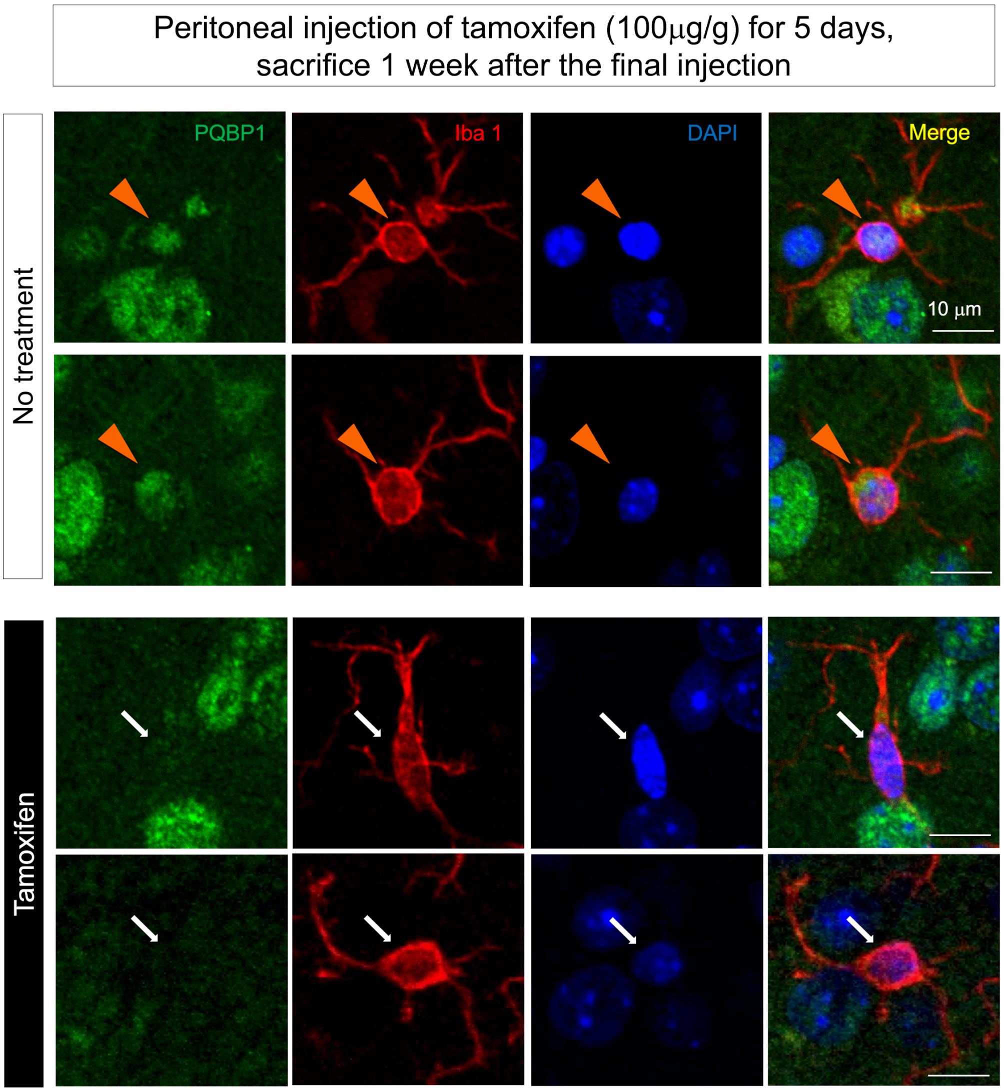

Supplementary Figure 5
PQBP1 is depleted by tamoxifen treatment
Pqbp1-cKO mice received peritoneal injection of tamoxifen (100 μg/g) for 5 days and were sacrificed 1 week after the final injection. Cerebral cortex tissues were stained with anti-PQBP1 and -Iba1 antibodies. Yellow arrowhead indicates microglia that express PQBP1 at high levels, while white arrow indicates microglia that express PQBP1 at low levels. The experiments were repeated independently three times with similar results.

Supplementary Figure 6

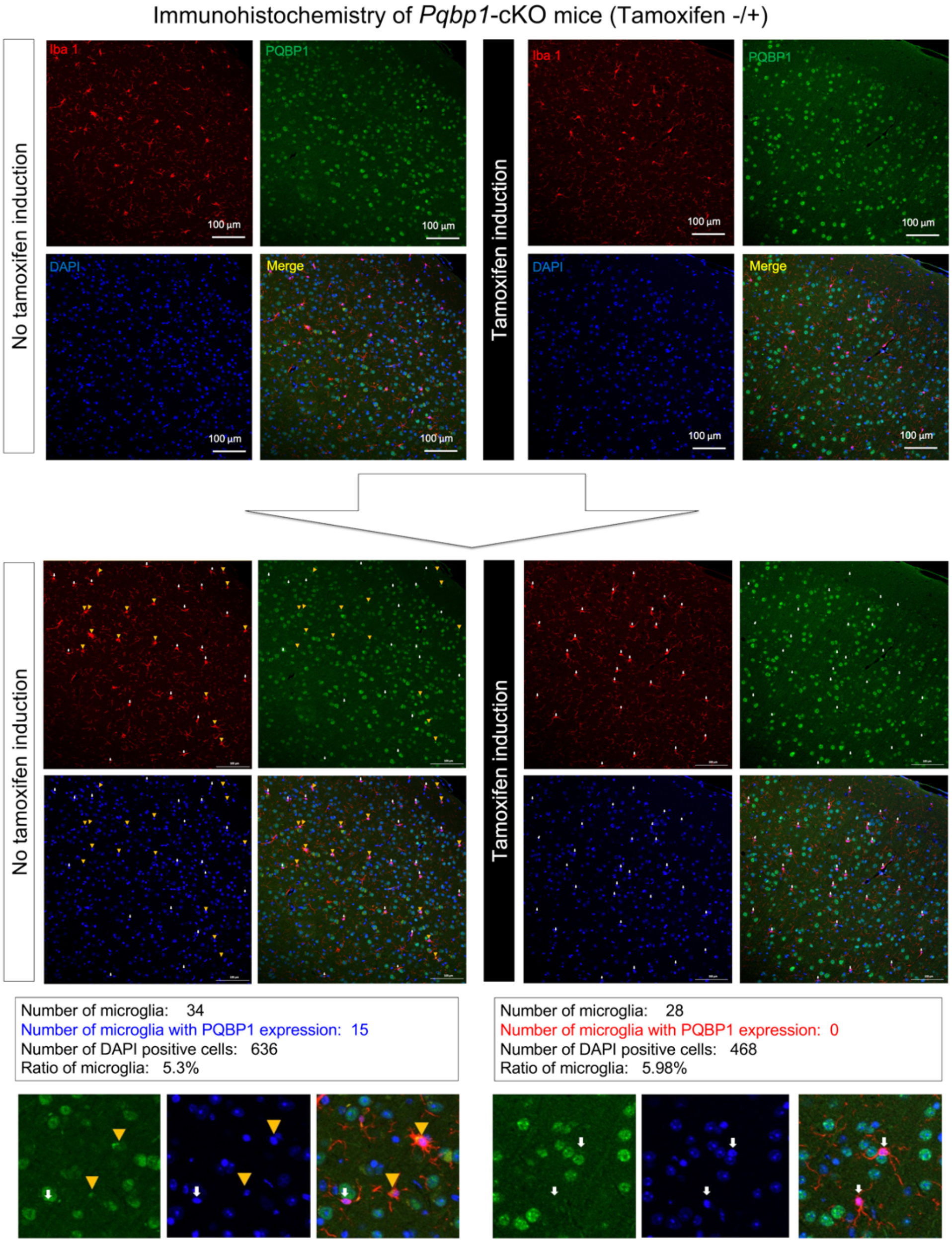

Supplementary Figure 6

Quantitative analysis of PQBP1 depletion by tamoxifen treatment

Low magnification images of cerebral cortex tissues of tamoxifen-induced *Pqbp1*-cKO mice taken by confocal microscopy (upper panels). Microglia with a DAPI positive nucleus were examined for expression of PQBP1 protein (middle panels). Under high power magnification, high expressers (orange arrow heads) and low expressers (white arrows) of PQBP1 were observed in microglia of non-induced *Pqbp1*-cKO. In tamoxifen-induced *Pqbp1*-cKO mice, all microglia were low expressers.

Supplementary Figure 7

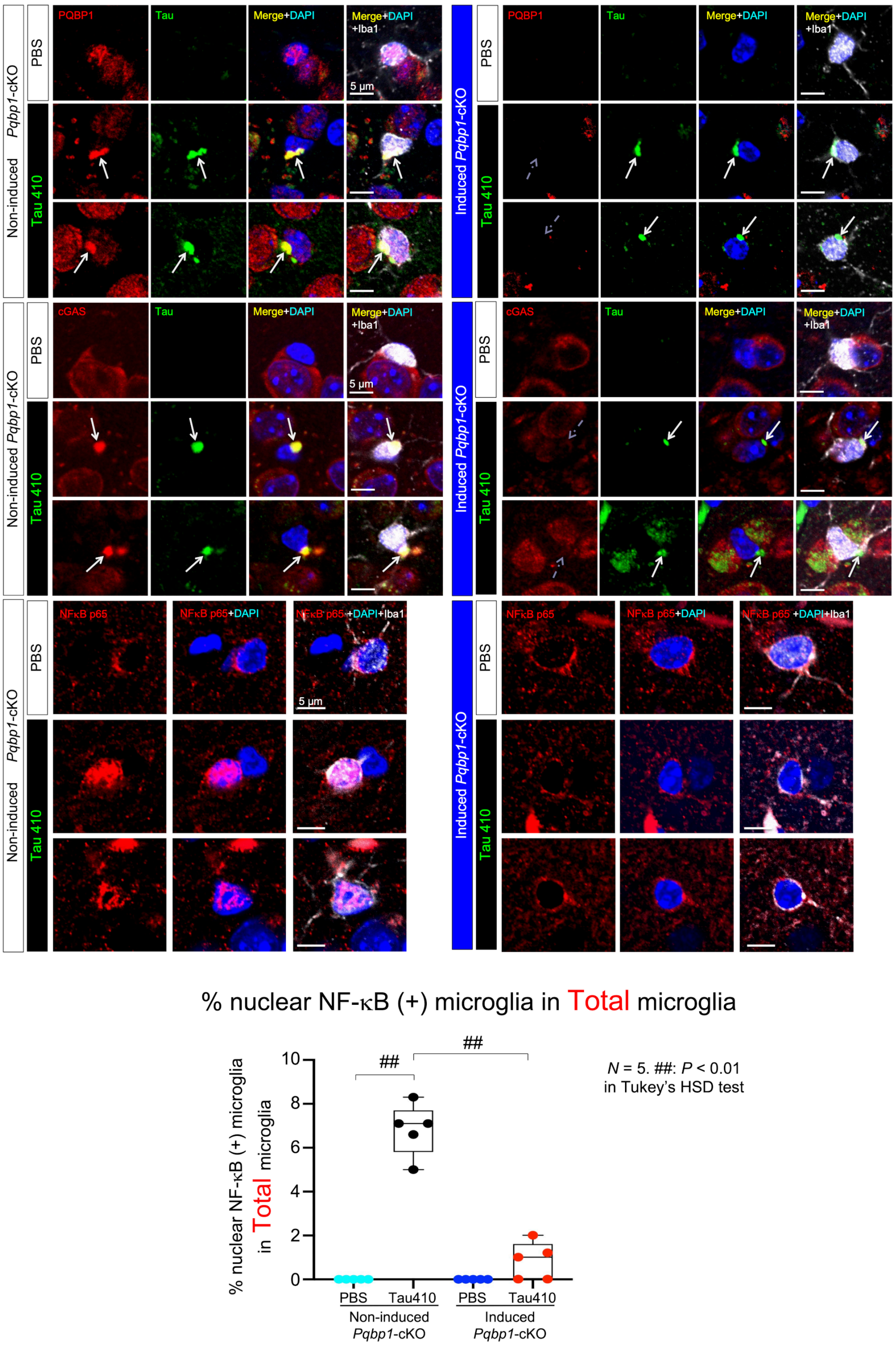

Supplementary Figure 7

PQBP1 is essential for in vivo activation of microglia by extrinsic Tau

Immunohistochemistry of Iba1-positive microglia with PQBP1+tau, cGAS+tau or NFκB p65 co-staining in injected area of non-induced *Pqbp1*-cKO (left panels) or tamoxifen-induced *Pqbp1*-cKO (right panels). Quantitative analysis of activated microglia (nuclear NFκB-positive) among total Iba1-positive microglia is shown in the lower graph. In the absence of *Pqbp1*, microglia activation was suppressed. $N = 5$ mice. $P = 5.87e^{-10}$ (Tau 410 / non-induced vs PBS / non-induced), $4.12e^{-9}$ (Tau 410 / non-induced vs Tau 410 / induced *Pqbp1*-cKO), ##: $P < 0.01$ in Tukey's HSD test. Box plots show the median, quartiles, and whiskers that represent data outside the 25th to 75th percentile range.

Supplementary Figure 8

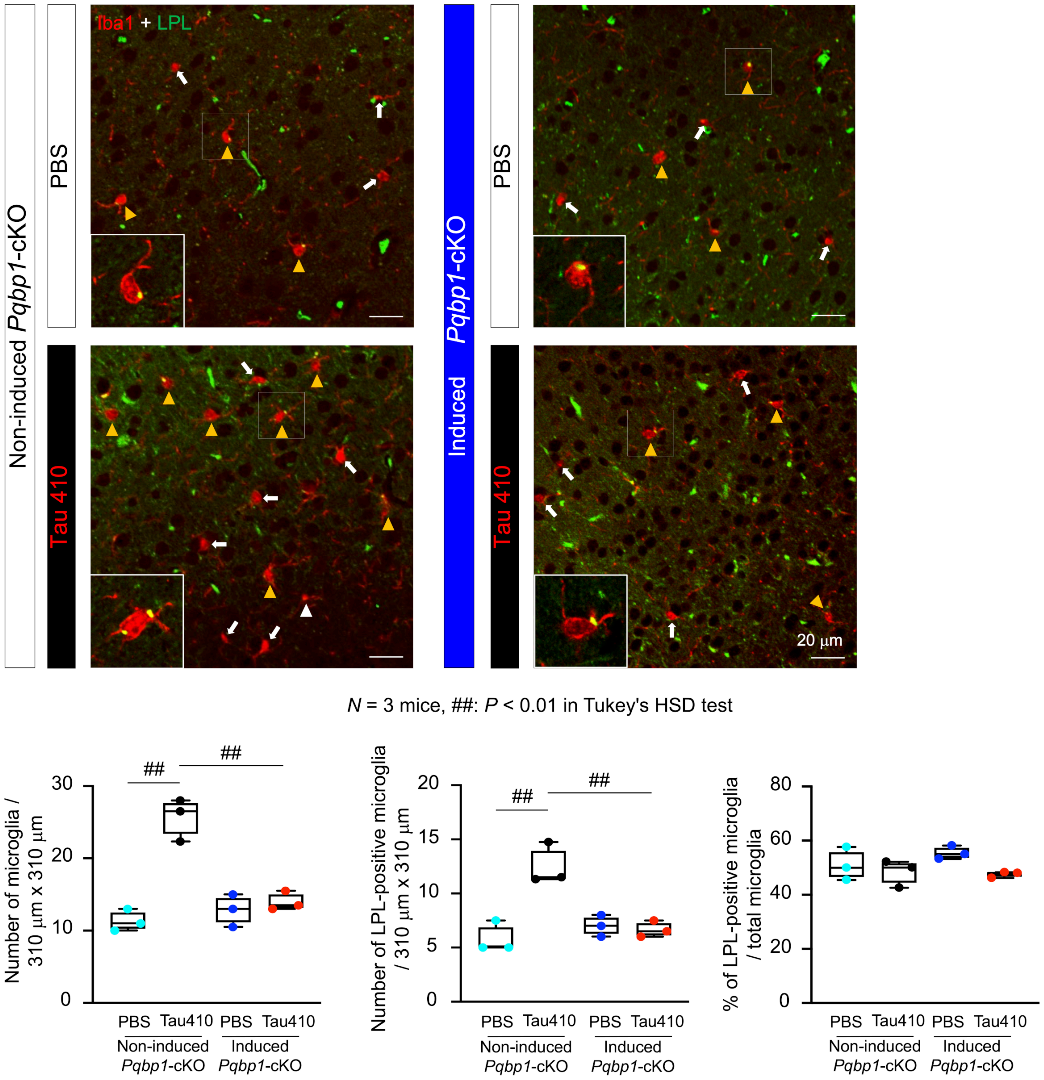

Supplementary Figure 8
Induction of LPL-positive microglia by Tau

LPL immunostaining revealing an increase of LPL-positive microglia in non-induced *Pqbp1*-cKO mice after injection of Tau410, but not in tamoxifen-induced *Pqbp1*-cKO mice lacking PQBP1 in microglia. Lower graphs show total number of microglia, number of LPL-positive microglia, and percentage of LPL-positive microglia in four groups. $N = 3$. Microglia number: P = 0.0002 (Tau 410 / non-induced vs PBS / non-induced), 0.0007 (Tau 410 / non-induced vs Tau 410 / induced *Pqbp1*-cKO). Number of LPL(+) microglia: P = 0.0014 (Tau 410 / non-induced vs PBS / non-induced), 0.0033 (Tau 410 / non-induced vs Tau 410 / induced *Pqbp1*-cKO). % LPL(+) microglia in total microglia: P = 0.8538 (Tau 410 / non-induced vs PBS / non-induced), 0.9956 (Tau 410 / non-induced vs Tau 410 / induced *Pqbp1*-cKO). ##: $P < 0.01$ in Tukey's HSD test. Box plots show the median, quartiles, and whiskers that represent data outside the 25th to 75th percentile range.

Supplementary Figure 9

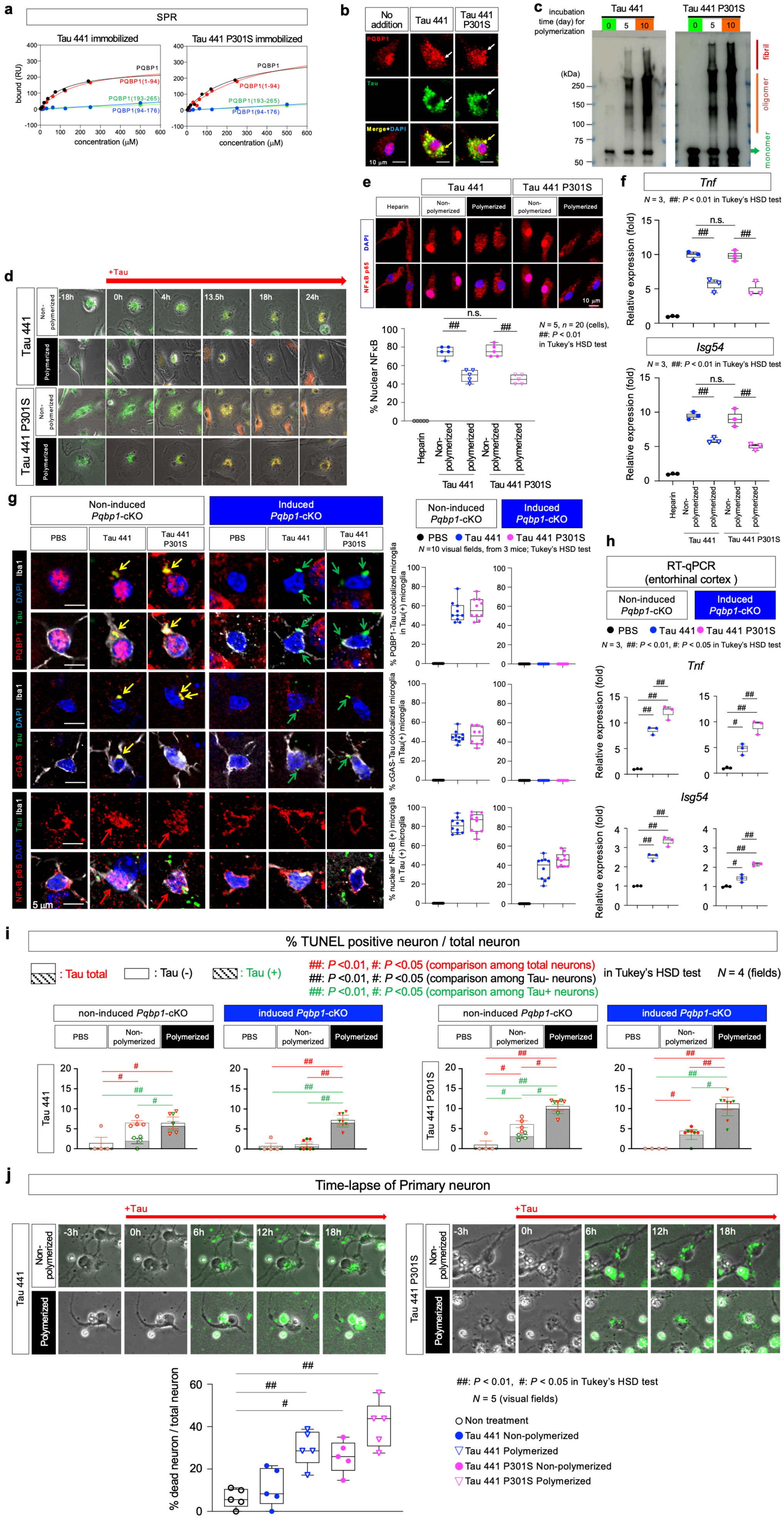

Supplementary Figure 9
Disease-linked mutation of Tau affects neurons but not microglia
a) SPR analyses of Tau 441 and Tau 441 P301S.
b) Immunocytochemistry of Tau-incorporated microglia shows similar colocalization of PQBP1 and Tau 441 or Tau 441 P301S in cytoplasmic foci. The experiments were repeated four times with similar results.
c) Polymerization of Tau 441 and Tau 441 P301S by incubation at 37 °C. The experiments were repeated four times with similar results.
d) Time-lapse imaging of microglia after addition of Tau to the culture medium revealed similar dynamism of colocalization between PQBP1 and Tau 441 or Tau 441 P301S.
e) Similar induction of nuclear translocation of NFκB in microglia by Tau 441 and Tau 441 P301S. $N = 5$, $n = 20$ cells. ##: $P < 0.01$ in Tukey's HSD test.
f) RT-qPCR revealed similar inductions of pro-inflammatory gene by addition of Tau 441 and Tau 441 P301S to primary microglia. $N = 3$. ##: $P < 0.01$ in Tukey's HSD test.
g) Immunohistochemistry revealed similar colocalization of Tau 441 and Tau 441 P301S with PQBP1 (upper image panels) or cGAS (middle image panels) at cytoplasmic foci and similar induction of nuclear NFκB (lower image panels) in the presence of PQBP1 in microglia (non-induced *Pqbp1*-cKO), which were suppressed by PQBP1 depletion (induced *Pqbp1*-cKO). Right graphs show percentage of tau-PQBP1 colocalized (upper), Tau-cGAS (middle) colocalized or nuclear NFκB-positive (lower) Iba1-positive microglia among all Iba1- and Tau-positive microglia. $N = 10$ visual fields from 3 mice. For statistical test, Tukey's HSD test was performed.
h) RT-qPCR based quantification of *Tnf* and *Isg54* mRNA in entorhinal cortex tissues of non-induced or induced *Pqbp1*-cKO mice after injection of PBS, Tau 441, or Tau 441 P301S. $N = 3$. #: $P < 0.05$, ##: $P < 0.01$ in Tukey's HSD test.
i) TUNEL-positive and NeuN-positive neurons were counted in entorhinal cortex tissues of non-induced or induced *Pqbp1*-cKO mice after injection of PBS, Tau 441 or Tau 441 P301S in non-polymerized or polymerized state. Values in each group are presented as mean ± SEM. $N = 4$ visual fields. #: $P < 0.05$, ##: $P < 0.01$ in Tukey's HSD test.
j) Neuronal death was observed by time-lapse imaging (upper images) and quantified (lower graph) in primary culture neurons after addition of Tau 441 or Tau 441 P301S in non-polymerized or polymerized state. H, hour. $N = 5$ visual fields. #: $P < 0.05$, ##: $P < 0.01$ in Tukey's HSD test.
Box plots show the median, quartiles, and whiskers that represent data outside the 25th to 75th percentile range.

Supplementary Figure 10

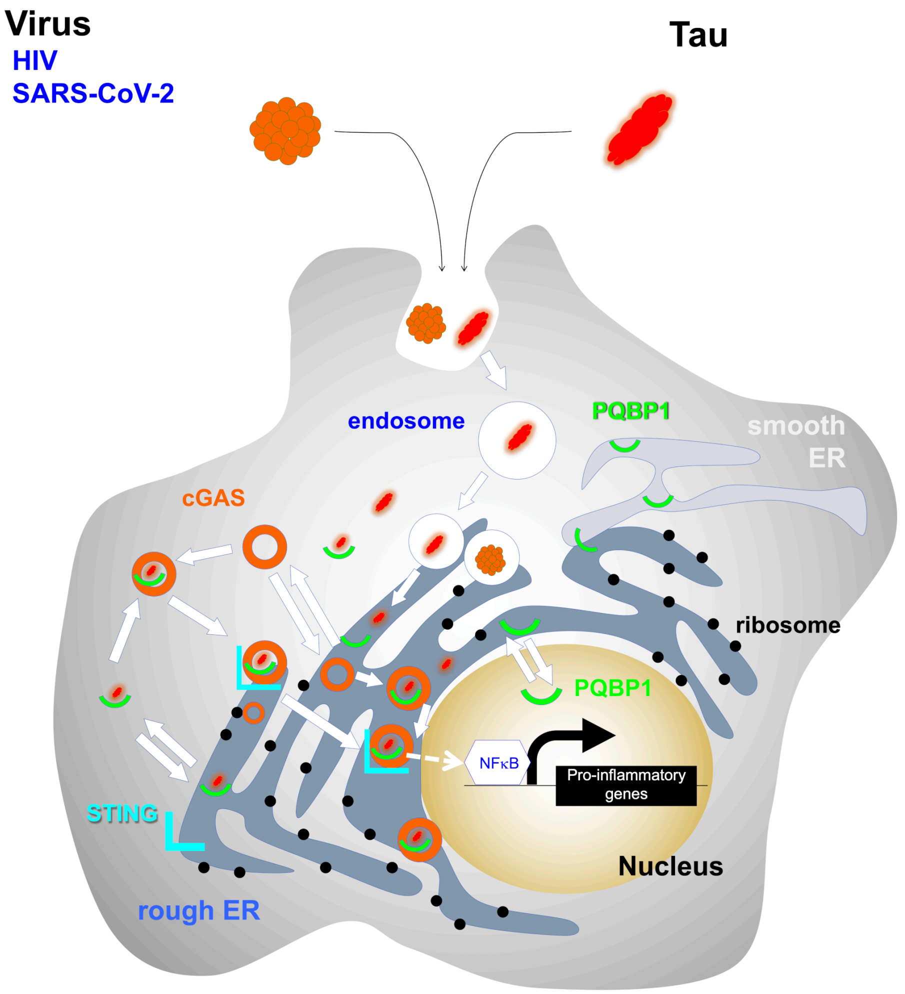

Supplementary Figure 10
Tau sensing by PQBP1-cGAS-STING pathway in microglia
Hypothetical scheme of the results of this study. ER, endoplasmic reticulum; SARS-CoV-2, subacute respiratory syndrome corona virus 2.

Supplementary Figure 11

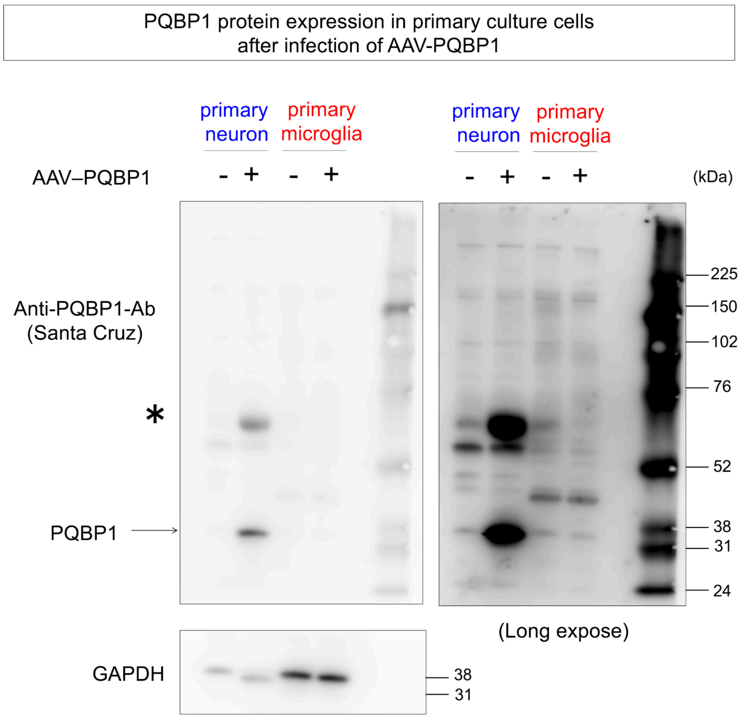

Supplementary Figure 11
AAV-PQBP1 increases PQBP1 in neurons but not in microglia
Cortical neurons and microglia were prepared from mice at E17 and P3, respectively, and were used for infection with AAV-PQBP1 (MOI 5,000). Western blot was performed at 5 days after infection.

Supplementary Table 1

K_d value of SPR in Figure 1b

K_d (M)					
	Tau 410	Tau 441	Tau 410 P179A	Tau 410 P216A	Tau 410 P179A/P216A
PQBP1 (1-265)	4.33×10^{-8}	1.22×10^{-8}	4.82×10^{-6}	3.14×10^{-7}	2.60×10^{-5}
PQBP1 (1–94)	7.69×10^{-8}	1.10×10^{-8}	6.34×10^{-6}	4.45×10^{-7}	7.33×10^{-5}
PQBP1 (94–176)	NB	NB	NB	NB	NB
PQBP1 (193–265)	NB	NB	NB	NB	NB

Supplementary Table 2

K_d value of SPR in Supplementary Figure 9a

K_d (M)		
	Tau 441	Tau 441 P301S
PQBP1 (1-265)	4.21×10^{-8}	5.15×10^{-8}
PQBP1 (1–94)	5.47×10^{-8}	4.62×10^{-8}
PQBP1 (94–176)	NB	NB
PQBP1 (193–265)	NB	NB

Supplementary Table 3

Primer list

#	Type	Primer	Sequence (5' - 3')
1	Mutagenesis	Tau410 P179A_Fw	CCCGCGGCTAAAACCCCACCATCCTCT
2	Mutagenesis	Tau410 P179A_Rev	CGGTTTTGCGGAGGGCGCCGATTTTGG
3	Mutagenesis	Tau410 P216A_Fw	AGCCTGGCAACACCACCGACCCGTGAA
4	Mutagenesis	Tau410 P216A_Rev	AGTGCATGAGGCTCGGACCGTTGTGGT
5	RT-qPCR	*Tnf*_Fw	TGCTTGTTGACAGCGGTCC
6	RT-qPCR	*Tnf* _Rev	ACTGGCCATCGTGGAGGTAC
7	RT-qPCR	*Isg54*_Fw	AGCAAGATGCACCAAGATGA
8	RT-qPCR	*Isg54*_Rev	CTGTGTCAAAGCGCTCAAAG
9	RT-qPCR	*Ifnβ*_Fw	GCCTTTGCCATCCAAGAGATGC
10	RT-qPCR	*Ifnβ*_Rev	ACACTGTCTGCTGGTGGAGTTC
11	RT-qPCR	*Cxcl10*_Fw	ATCATCCCTGCGAGCCTATCCT
12	RT-qPCR	*Cxcl10*_Rev	GACCTTTTTTGGCTAAACGCTTTC
13	RT-qPCR	*Gapdh*_Fw	TGAACGGGAAGCTCACTGG
14	RT-qPCR	*Gapdh*_Rev	TCCACCACCCTGTTGCTGTA

Supplementary Table 4

List of antibodies

#	Antibody	Company & catalog number	Dilution	Manufacturer's website
1	rabbit anti-PQBP1 FL265	Santa Cruz Biotechnology, sc-32910	Quantitative IP 1:80<br>ICC 1:250	https://datasheets.scbt.com/sds/aghs/en/sc-32910.pdf#
2	rabbit anti-PQBP1	Bethyl, A302-801A	IP/IHC 1:200<br>ICC 1:150<br>WB 1:1000	https://www.bethyl.com/product/A302-801A/PQBP1+Antibody
3	mouse anti-PQBP1	Santa Cruz Biotechnology,sc-374260	ICC/IHC 1:200	https://datasheets.scbt.com/sc-374260.pdf
4	mouse anti-Tau	Merck, MAB361	Quantitative IP 1:400<br>WB 1:3000	https://www.sigmaaldrich.com/JP/ja/product/mm/mab361
5	mouse anti-Tau	Abcam, ab80579	IP 1:200<br>WB 1:10,000	https://www.abcam.com/tau-antibody-tau-5-bsa-and-azide-free-ab80579.html
6	mouse anti-tau	Thermo Fisher Scientific, MA5-15108	IHC 1:500	https://www.thermofisher.com/antibody/product/Tau-Antibody-clone-S-125-0-Monoclonal/MA5-15108
7	mouse anti-phospho-tau(AT-8)	Innogenetics, 90206	ICC/IHC 1:1000	https://search.cosmobio.co.jp/cosmo_search_p/search_gate2/docs/IGT_/90206.20190605.pdf
8	rabbit anti-Iba1	WAKO, 019-19741	ICC/IHC 1:1000	https://labchem-wako.fujifilm.com/us/product/detail/W01W0101-1974.html
9	goat anti-Iba1	WAKO, 011-27991	IHC 1:500	https://labchem-wako.fujifilm.com/us/product/detail/W01W0101-2799.html
10	goat anti-Iba1	Abcam,ab107159	IHC 1:500	https://www.abcam.com/iba1-antibody-ab107159.html
11	human IgG	Thermo Fisher Scientific,12000C	Quantitative IP 1:400	https://www.thermofisher.com/antibody/product/Human-IgG-Isotype-Control/12000C
12	anti-cGAS rabbit antibody	Merck, ABF124	ICC/IHC 1:500<br>WB 1:1000	https://www.merckmillipore.com/JP/en/product/Anti-cGAS-Antibody,MM_NF-ABF124
13	mouse-anti-TREM2	Santa Cruz Biotechnology, sc-373828	ICC 1:100	https://datasheets.scbt.com/sc-373828.pdf
14	mouse anti-LRP1 antibody	Santa Cruz Biotechnology, sc-57353	ICC 1:250	https://datasheets.scbt.com/sc-57353.pdf
15	rabbit anti-NFκB p65 (C-20)	Santa Cruz Biotechnology, sc-372	ICC/IHC 1:250	https://datasheets.scbt.com/sc-372.pdf
16	mouse anti-LPL	Abcam, ab21356	IHC 1:100	https://www.abcam.com/lipoprotein-lipase-antibody-lpla4-ab21356.html
17	rabbit anti-MAP2	Abcam, ab32454	IHC 1:1000	https://www.abcam.com/map2-antibody-neuronal-marker-ab32454.html
18	mouse anti-GFAP-Cy3	Sigma aldrich, C9205	IHC 1:5000	https://www.sigmaaldrich.com/JP/ja/product/sigma/c9205?context=product
19	mouse anti-NeuN	Abcam, ab104224	IHC 1:1000	https://www.abcam.com/neun-antibody-1b7-neuronal-marker-ab104224.html
20	Biotin-16-dUTP	Roche, 11093070910	IHC 1:100	http://www.qcbio.com/roche/Biotin-16-dUTP.asp
21	Terminal Transferase	Roche, 03333574001	IHC 1:100	https://custombiotech.roche.com/home/Product_Details/3_6_14_3_7_2.html
22	rabbit anti-STING	Cell Signaling Technology, 13647S	WB 1:3000	https://www.cellsignal.com/products/primary-antibodies/sting-d2p2f-rabbit-mab/13647
23	rabbit anti-phospho-Ser536-NFκB	Cell Signaling Technology, 3033S	WB 1:1000	https://www.cellsignal.com/products/primary-antibodies/phospho-nf-kb-p65-ser536-93h1-rabbit-mab/3033
24	rabbit anti-phospho-Ser396-IRF3	Cell Signaling Technology, 4947S	WB 1:1000	https://www.cellsignal.com/products/primary-antibodies/phospho-irf-3-ser396-4d4g-rabbit-mab/4947
25	mouse anti-GAPDH	Millipore, MAB374	WB 1:5000	https://www.sigmaaldrich.com/JP/ja/product/MM/MAB374

Supplementary Table 5

Mouse information

#	Strain	Age	Sex	Relatived Figure
1	*Pqbp1*-cKO: *Cx3cr1*$^{CreER/CreER}$/*Pqbp1*$^{floxX/Y}$ (C57BL/6 background)	8 weeks	male	Figure 6b,c,d; Figure 7c,d,e,f; Figure 9a,b; Figure 10a; Supplementary Figure 5,6,7,8,9g,9h,9i
2	B6.129P2(Cg)-*Cx3cr1*^{tm1Litt}/J (*Cx3cr-1*^{GFP})	8 weeks	male	Figure 6e,f
3	R6/2	12 weeks	male	Supplementary Figure 2
