# Supplementary material for: Tau activates microglia via the PQBP1-cGAS-STING pathway to promote brain inflammation
Source: Nat Commun. 2021 Nov 15;12:6565. doi: 10.1038/s41467-021-26851-2 (PMC8592984; doi:10.1038/s41467-021-26851-2)
Supplement: Supplementary file 3 — Description of Additional Supplementary Files [file 41467_2021_26851_MOESM3_ESM.pdf]

Description of Additional Supplementary Files

File Name: Supplementary Movie 1

Description: **Live imaging of PQBP1-EGFP and non-polymerized Tau410 in primary normal microglia**

PQBP1-EGFP proteins in ER were distributed as green dots around the nucleus of microglia. At 0 hour, non-polymerized TAMRA-labeled Tau 410 was added to the medium, which was taken up into the cytoplasm of microglia and distributed homogeneously. From 3 h, TAMRA-labeled Tau 410 began to merge with dots of PQBP1-EGFP and was distributed more diffusely in the cell. Interestingly, a small number of microglia did not incorporate TAMRA-labeled Tau 410 and remained green.

File Name: Supplementary Movie 2

Description: **Live imaging of PQBP1-EGFP and polymerized Tau441 in primary normal microglia**

At 0 hour, polymerized TAMRA-labeled Tau 441 was added to the medium. After 30 minutes, Tau 441 polymer moved to the peri-nuclear region and pushed out PQBP1-EGFP to peripheral ER.

File Name: Supplementary Movie 3

Description: **Live imaging of PQBP1-EGFP and non-polymerized Tau 410 P216A in primary normal microglia**

At 0 hour, non-polymerized TAMRA-labeled Tau 410 carrying the P216A mutation was added to the medium. After 4 hours, Tau 410 P216A appears in a part of microglia. However, Tau 410 P216A might have a tendency to accumulate in low expressers of PQBP1-EGFP, and red signals of Tau 410 P216A did not increase remarkably in high expressers of PQBP1-EGFP. Eventually, merging tended to be delayed between PQBP1 and the binding mutants of Tau in ER, and merging started from 8 hours after addition of Tau 410 P216A.
