# Supplementary material for: Tau activates microglia via the PQBP1-cGAS-STING pathway to promote brain inflammation
Source: Nat Commun. 2021 Nov 15;12:6565. doi: 10.1038/s41467-021-26851-2 (PMC8592984; doi:10.1038/s41467-021-26851-2)
Supplement: Supplementary file 7 — Reporting Summary [file 41467_2021_26851_MOESM7_ESM.pdf]

nature portfolio

Corresponding author(s): Hitoshi Okazawa

Last updated by author(s): Sep 21, 2021

Reporting Summary

Nature Portfolio wishes to improve the reproducibility of the work that we publish. This form provides structure for consistency and transparency in reporting. For further information on Nature Portfolio policies, see our Editorial Policies and the Editorial Policy Checklist.

Statistics

For all statistical analyses, confirm that the following items are present in the figure legend, table legend, main text, or Methods section.

n/a	Confirmed	
☐	☒	The exact sample size (n) for each experimental group/condition, given as a discrete number and unit of measurement
☐	☒	A statement on whether measurements were taken from distinct samples or whether the same sample was measured repeatedly
☐	☒	The statistical test(s) used AND whether they are one- or two-sided<br>*Only common tests should be described solely by name; describe more complex techniques in the Methods section.*
☐	☒	A description of all covariates tested
☐	☒	A description of any assumptions or corrections, such as tests of normality and adjustment for multiple comparisons
☐	☒	A full description of the statistical parameters including central tendency (e.g. means) or other basic estimates (e.g. regression coefficient) AND variation (e.g. standard deviation) or associated estimates of uncertainty (e.g. confidence intervals)
☐	☒	For null hypothesis testing, the test statistic (e.g. F, t, r) with confidence intervals, effect sizes, degrees of freedom and P value noted<br>*Give P values as exact values whenever suitable.*
☒	☐	For Bayesian analysis, information on the choice of priors and Markov chain Monte Carlo settings
☒	☐	For hierarchical and complex designs, identification of the appropriate level for tests and full reporting of outcomes
☒	☐	Estimates of effect sizes (e.g. Cohen's d, Pearson's r), indicating how they were calculated

Our web collection on statistics for biologists contains articles on many of the points above.

Software and code

Policy information about availability of computer code

Data collection	No software was used.
Data analysis	GraphPad Prism 8 software (GraphPad Software, La Jolla, CA, USA) was used for statistical analyses and to plot data.<br>R version 3.6.2 (The R Foundation for Statistical Computing) was used for statistical analyses.<br>Microsoft Excel for Microsoft 365 was used to draw graphs.

For manuscripts utilizing custom algorithms or software that are central to the research but not yet described in published literature, software must be made available to editors and reviewers. We strongly encourage code deposition in a community repository (e.g. GitHub). See the Nature Portfolio guidelines for submitting code & software for further information.

Data

Policy information about availability of data

All manuscripts must include a data availability statement. This statement should provide the following information, where applicable:

- Accession codes, unique identifiers, or web links for publicly available datasets
- A description of any restrictions on data availability
- For clinical datasets or third party data, please ensure that the statement adheres to our policy

We wrote the following sentences in "Data Availability" section.
All relevant data are available within the article and its Supplementary Information files. Source data are provided with this paper. A reporting summary for this article is available as a Supplementary Information file.
UniProt database of subcellular locations was used for Sting (https://www.uniprot.org/uniprot/Q86WV6) and for cGAS (https://www.uniprot.org/uniprot/Q8N884).
Human FUS, TDP43, hnRNPA1 and hnRNPA2B1 protein sequences were retrieved from Uniprot (https://www.uniprot.org/).

Field-specific reporting

Please select the one below that is the best fit for your research. If you are not sure, read the appropriate sections before making your selection.

☒ Life sciences ☐ Behavioural & social sciences ☐ Ecological, evolutionary & environmental sciences

For a reference copy of the document with all sections, see nature.com/documents/nr-reporting-summary-flat.pdf

Life sciences study design

All studies must disclose on these points even when the disclosure is negative.

Sample size	We performed power analysis to estimate the required sample size (n) for each of experiments.
Data exclusions	There are no exclusion criteria for all analysis.
Replication	Experiments were independently repeated, the numbers of biological replicates are presented in the Figures.
Randomization	The selection of animals and the behavior analyses were performed by independent researchers.<br>Animals were allocated into experimental groups at random.<br>The selection of images from immunohistochemistry/immunocytochemisty and the actual experiments of IHC/ ICC were done by different researchers. In vitro live-cell imaging were done by different researchers.<br>Western blots are repeated until the necessary N was acquired.
Blinding	The information about group allocation or samples were opened to the data analyst or image acquisition researchers after finalizing results (m ake graphs etc).

Reporting for specific materials, systems and methods

We require information from authors about some types of materials, experimental systems and methods used in many studies. Here, indicate whether each material, system or method listed is relevant to your study. If you are not sure if a list item applies to your research, read the appropriate section before selecting a response.

Materials & experimental systems

n/a	Involved in the study	
☐	☒	Antibodies
☒	☐	Eukaryotic cell lines
☒	☐	Palaeontology and archaeology
☐	☒	Animals and other organisms
☒	☐	Human research participants
☒	☐	Clinical data
☒	☐	Dual use research of concern

Methods

n/a	Involved in the study	
☒	☐	ChIP-seq
☒	☐	Flow cytometry
☒	☐	MRI-based neuroimaging

Antibodies

Antibodies used

All antibodies used in the study are listed in the method and in supplementary table 4.

The antibodies used for immunohistochemistry were dilution as following, mouse anti-PQBP1 (1:200, sc-374260, Santa Cruz Biotechnology, Dallas, TX, USA); rabbit anti-PQBP1 (1:200, A302-801A, Bethyl Laboratory, Montgomery, TX, USA); rabbit anti-Iba1 (1:1000, 019-19741, WAKO, Osaka, Japan); goat anti-Iba1 (1:500, 011-27991, WAKO, Osaka, Japan); goat anti-Iba1 (1:500, ab107159, Abcam, Cambridge, UK); rabbit anti-NFkappaB p65 (C-20) (1:250, sc-372, Santa Cruz Biotechnology, Dallas, TX, USA); mouse anti-phospho-tau (1:1000, 90206, Innogenetics, Ghent, Belgium); mouse anti-tau (1:500, MA5-15108, Thermo Fisher Scientific, Waltham, MA, USA); rabbit anti-cGAS (1:500, ABF124, Merck, Darmstadt, Germany); mouse anti-LPL (1:100, ab21356, Abcam, Cambridge, UK); rabbit anti-MAP2 (1:1000, ab32454, Abcam, Cambridge, UK); mouse anti-GFAP-Cy3 (1:5000, C9205, Sigma aldrich, St. Louis, MO, USA); mouse anti-NeuN (1:1000, ab104224, Abcam, Cambridge, UK). Secondary antibodies were as follows: donkey anti-goat IgG Alexa568 (1:1000, A11057, Molecular Probes, Eugene, OR, USA); donkey anti-mouse IgG Alexa488 (1:1000, A21202, Molecular Probes, Eugene, OR, USA); donkey anti-rabbit IgG Alexa647 (1:1000, A31573, Molecular Probes, Eugene, OR, USA). Nuclei were stained with DAPI (0.2 microg/ml in PBS, D523, DOJINDO Laboratories, Kumamoto, Japan).
For nick-end-labeling, frozen sections were washed three times with PBS containing 0.1% Tween-20 (PBST) at room temperature. The sections were incubated with labeling reaction mix [Biotin-16-dUTP (11093070910, Roche, Mannheim, Germany) and Terminal Transferase (03333574001, Roche, Mannheim, Germany)] at 37°C for 2 hours, washed with PBST three times, and then incubated with Alexa Fluor 633–conjugated streptavidin (S21375, Thermo Fisher Scientific) at room temperature for 1 hour.

The antibodies used for immunocytochemistry ("Immunocytochemistry" and "Knockout of LRP1 and TREM2 in primary microglia") were diluted as follows: anti-PQBP1 antibody (1:250, FL-265, Santa Cruz Biotechnology, Dallas, TX, USA), anti-NFkappaB p65 Antibody

(C-20) (1:250, sc-372, Santa Cruz Biotechnology, Dallas, TX, USA), anti-phospho-tau antibody (1:1000, 90206, Innogenetics, Ghent, Belgium), anti-cGAS rabbit antibody (1:500, ABF124, Merck, Darmstadt, Germany); mouse-anti-TREM2 (1:100, sc-373828, Santa Cruz Biotechnology, Dallas, TX, USA); rabbit-anti-PQBP1(1:150, Bethyl Laboratories, A302-801A, Montgomery,TX, USA)); Cy5-conjugated anti-mouse IgG (1:500, 715-175-151, Jackson Laboratory, Bar Harbor, ME, USA); and Alexa Fluor 405–conjugated anti-rabbit IgG (1:1000, A48258, Molecular Probes, Eugene, OR, USA). For multiple co-staining , mouse anti-LRP1 antibody (1:250, sc-57353, Santa Cruz Biotechnology, Dallas, TX, USA) was labeled by Zenon Secondary Detection-Based Antibody Labeling Kits (Zenon™ Alexa Fluor™ 555 Rabbit IgG Labeling Kit, Z-25305, Thermo Fisher Scientific, Waltham, MA, USA)

The antibodies used for immunoprecipitation were diluted as follows: rabbit anti-PQBP1 (1:80, sc-32910, FL-265, Santa Cruz Biotechnology, Dallas, TX, USA), mouse anti-Tau (1:400, MAB361, Merck, Darmstadt, Germany), human IgG (1:400, 12000C, Thermo Fisher Scientific, Waltham, MA, USA), rabbit anti-PQBP1 antibody (1:200, A302-801A, Bethyl, Montgomery, TX, USA), and mouse anti-Tau antibody (1:200, ab80579, Abcam, Cambridge, UK).

The antibodies used for western blotting were diluted as follows: mouse anti-Tau (1:3000, MAB361, Millipore, Burlington, MA, USA), rabbit anti-PQBP1 (1:500, sc-32910, Santa Cruz Biotechnology, Dallas, TX, USA); rabbit anti-cGAS (1:1000, ABF124, Merck, Darmstadt, Germany); rabbit anti-STING (1:3000, 13647S, Cell Signaling Technology, Danvers, MA, USA); rabbit anti-phospho-Ser536-NF B (1:1000, 3033S, Cell Signaling Technology, Danvers, MA, USA), rabbit anti-phospho-Ser396-IRF3 (1:1000, 4947S, Cell Signaling Technology, Danvers, MA, USA); mouse anti-GAPDH (1:5000, MAB374, Millipore, Burlington, MA, USA); mouse anti-Tau (1:10,000, ab80579, Abcom, Cambridge, UK); rabbit anti-PQBP1 (1:1000, A302-801A, Bethyl, Montgomery, TX, USA); HRP-conjugated anti-mouse IgG (1:3000, NA931VA, GE Healthcare, Chicago, IL, USA); and HRP-conjugated anti-rabbit IgG (1:3000, NA934VS, GE Healthcare, Chicago, IL, USA).

Validation

We added the following information in supplementary table 4.
Rabbit anti-PQBP1 FL265 (Santa Cruz Biotechnology, sc-32910, https://datasheets.scbt.com/sds/aghs/en/sc-32910.pdf#); rabbit anti-PQBP1 (Bethyl, A302-801Ahttps://www.bethyl.com/product/A302-801A/PQBP1+Antibody); mouse anti-PQBP1(Santa Cruz Biotechnology,sc-374260, https://datasheets.scbt.com/sc-374260.pdf); mouse anti-Tau (Merck, MAB361, https://www.sigmaaldrich.com/JP/ja/product/mm/mab361); mouse anti-Tau (Abcam, ab80579, https://www.abcam.com/tau-antibody-tau-5-bsa-and-azide-free-ab80579.html); mouse anti-tau (Thermo Fisher Scientific, MA5-15108, https://www.thermofisher.com/antibody/product/Tau-Antibody-clone-S-125-0-Monoclonal/MA5-15108); mouse anti-phospho-tau(AT-8)(Innogenetics, 90206, https://search.cosmobio.co.jp/cosmo_search_p/search_gate2/docs/IGT_/90206.20190605.pdf); rabbit anti-Iba1 (WAKO, 019-19741,https://labchem-wako.fujifilm.com/us/product/detail/W01W0101-1974.html); goat anti-Iba1 (WAKO, 011-27991, https://labchem-wako.fujifilm.com/us/product/detail/W01W0101-2799.html); goat anti-Iba1 (Abcam, ab107159, https://www.abcam.com/iba1-antibody-ab107159.html); human IgG (Thermo Fisher Scientific,12000C, https://www.thermofisher.com/antibody/product/Human-IgG-Isotype-Control/12000C); rabbit anti-cGAS rabbit antibody (Merck, ABF124, https://www.merckmillipore.com/JP/en/product/Anti-cGAS-Antibody,MM_NF-ABF124); mouse anti-TREM2 (Santa Cruz Biotechnology, sc-373828, https://datasheets.scbt.com/sc-373828.pdf); mouse anti-LRP1 antibody (Santa Cruz Biotechnology, sc-57353, https://datasheets.scbt.com/sc-57353.pdf); rabbit anti-NFkB p65 (C-20) (Santa Cruz Biotechnology, sc-372, https://datasheets.scbt.com/sc-372.pdf); mouse anti-LPL (Abcam, ab21356, https://www.abcam.com/lipoprotein-lipase-antibody-lpla4-ab21356.html); rabbit anti-MAP2 (Abcam, ab32454, https://www.abcam.com/map2-antibody-neuronal-marker-ab32454.html); mouse anti-GFAP-Cy3 (Sigma aldrich, C9205,https://www.sigmaaldrich.com/JP/ja/product/sigma/c9205?context=product); mouse anti-NeuN (Abcam, ab104224, https://www.abcam.com/neun-antibody-1b7-neuronal-marker-ab104224.html); Biotin-16-dUTP (Roche, 11093070910, http://www.qcbio.com/roche/Biotin-16-dUTP.asp); Terminal Transferase (Roche, 03333574001, https://custombiotech.roche.com/home/Product_Details/3_6_14_3_7_2.html); rabbit anti-STING (Cell Signaling Technology, 13647S, https://www.cellsignal.com/products/primary-antibodies/sting-d2p2f-rabbit-mab/13647); rabbit anti-phospho-Ser536-NFkB (Cell Signaling Technology, 3033S, https://www.cellsignal.com/products/primary-antibodies/phospho-nf-kb-p65-ser536-93h1-rabbit-mab/3033); rabbit anti-phospho-Ser396-IRF3 (Cell Signaling Technology, 4947S, https://www.cellsignal.com/products/primary-antibodies/phospho-irf-3-ser396-4d4g-rabbit-mab/4947); mouse anti-GAPDH (Millipore, MAB374, https://www.sigmaaldrich.com/JP/ja/product/MM/MAB374).

Animals and other organisms

Policy information about studies involving animals; ARRIVE guidelines recommended for reporting animal research

Laboratory animals

We added the housing condition in Method section (Animal housing condition) as follows: The mice were maintained at 22 °C, suitable humidity (typically 50%) and a 12-hour dark/light cycle. Detailed information such as age and sex of the mice is listed in Supplementary Information (Supplementary Table 5).

Wild animals

The study did not involve any wild animals.

Field-collected samples

The study did not involve any samples collected from the field.

Ethics oversight

Animal experiments were approved by the Committees on Gene Recombination Experiments and Animal Experiments of Tokyo Medical and Dental University (G2018-082C and A2019-218C2). The experiments using human samples were approved by the Committee on Human Ethics of the Tokyo Medical and Dental University (O2014-005-13/O2020-002).

Note that full information on the approval of the study protocol must also be provided in the manuscript.
